# Effectiveness of interventions to address obesity and health risk behaviours among people with severe mental illness in low- and middle-income countries (LMICs): a systematic review and meta analysis

**DOI:** 10.1017/gmh.2022.21

**Published:** 2022-06-22

**Authors:** Gerardo A. Zavala, Olamide Todowede, Papiya Mazumdar, Faiza Aslam, Asiful Haidar Choudhury, Alexander Jarde, Humaira Khalid, Sadananda Reddy, Simon Gilbody, Najma Siddiqi

**Affiliations:** 1 Department of Health Sciences, University of York, York, UK; 2 Institute of Psychiatry (IoP), Benazir Bhutto Hospital, Rawalpindi Medical University, Rawalpindi, Pakistan; 3 ARK Foundation, Dhaka, Bangladesh; 4 School of Social Sciences/Psychology, CHRIST (Deemed to be University), Bengaluru, India; 5 Hull York Medical School, York, UK

**Keywords:** Health risk behaviours, low and middle income countries, schizophrenia, severe mental illness

## Abstract

**Introduction:**

People with severe mental illness (SMI) are more likely to have obesity and engage in health risk behaviours than the general population. The aims of this study are (1) evaluate the effectiveness of interventions that focus on body weight, smoking cessation, improving sleeping patterns, and alcohol and illicit substance abuse; (2) Compare the number of interventions addressing body weight and health risk behaviours in low- and middle-income countries (LMICs) *v*. those reported in published systematic reviews focusing on high-income countries (HICs).

**Methods:**

Intervention studies published up to December 2020 were identified through a structured search in the following database; OVID MEDLINE (1946–December 2020), EMBASE (1974–December 2020), CINAHL (1975–2020), APA PsychoINFO (1806–2020). Two authors independently selected studies, extracted study characteristics and data and assessed the risk of bias. and risk of bias was assessed using the Cochrane risk of bias tool V2. We conducted a narrative synthesis and, in the studies evaluating the effectiveness of interventions to address body weight, we conducted random-effects meta-analysis of mean differences in weight gain. We did a systematic search of systematic reviews looking at cardiometabolic and health risk behaviours in people with SMI. We compared the number of available studies of LMICs with those of HICs.

**Results:**

We assessed 15 657 records, of which 9 met the study inclusion criteria. Six focused on healthy weight management, one on sleeping patterns and two tested a physical activity intervention to improve quality of life. Interventions to reduce weight in people with SMI are effective, with a pooled mean difference of −4.2 kg (95% CI −6.25 to −2.18, 9 studies, 459 participants, *I*^2^ = 37.8%). The quality and sample size of the studies was not optimal, most were small studies, with inadequate power to evaluate the primary outcome. Only two were assessed as high quality (i.e. scored ‘low’ in the overall risk of bias assessment). We found 5 reviews assessing the effectiveness of interventions to reduce weight, perform physical activity and address smoking in people with SMI. From the five systematic reviews, we identified 84 unique studies, of which only 6 were performed in LMICs.

**Conclusion:**

Pharmacological and activity-based interventions are effective to maintain and reduce body weight in people with SMI. There was a very limited number of interventions addressing sleep and physical activity and no interventions addressing smoking, alcohol or harmful drug use. There is a need to test the feasibility and cost-effectiveness of context-appropriate interventions to address health risk behaviours that might help reduce the mortality gap in people with SMI in LMICs.

## Introduction

People with severe mental illness (SMI) die on average 10–20 years earlier than the general population, and this mortality gap is even bigger in low- and middle-income countries (LMICs) (Liu *et al*., 2017). People with SMI are disproportionately affected by cardiometabolic risk factors, including obesity and long term physical health conditions that can be attributed to the presence of additional health risk behaviours such as smoking, poor diet, physical inactivity, poor sleep, harmful alcohol use and a side effect of antipsychotic medication (Scott and Happell, 2011; Bartlem *et al*., 2015). Numerous challenges interfere with achieving health risk modification in this population, including low or no access to healthcare and health risk modification advice, stigma, the impact of psychoactive medication on motivation, and poverty (Naslund *et al*., 2017).

Influential position statements by the WHO and a Lancet Commission have taken note of the importance of addressing cardiometabolic and health risk behaviours to improve physical health and reduce the mortality gap in people with SMI (WHO, 2018; Firth *et al*., 2019). Reviews evaluating interventions to address obesity, (Teasdale *et al*., 2017*a*, 2017*b*) smoking, (Peckham *et al*., 2017), alcohol abuse, (Boniface *et al*., 2018) and multiple health risk behaviours (Cabassa *et al*., 2010) in this population have found that interventions are effective for mitigating these factors in this population. There are no systematic reviews which focus on evidence from the perspective of LMICs, where trial based evidence is essential in formulating policy and practice, but evidence from high-income countries (HICs) may not necessarily be applicable (Cabassa *et al*., 2010). Research evidence from low-resourced health systems will be contextually useful and may have addressed the specific challenges of health improvement, behaviour change and prevention that this population is facing in LMICs.

We performed a systematic review and evidence synthesis of the available trial-based literature to identify important evidence gaps to inform the future research agenda. The aim of this review is to evaluate ‘what works’ in the modification or prevention of cardiometabolic and health risk behaviours that are detrimental to health or that promote behaviours that facilitate good health in people with SMI in LMICs. More specifically the review assessed the effectiveness of interventions that focus on (1) weight reduction; (2) smoking cessation; (3) improving sleeping patterns; and (4) alcohol and illicit substance abuse. As a secondary aim we compared the number of interventions addressing body weight and health risk behaviours in LMICs *v*. those reported in other published systematic reviews.

## Methods

The review was reported in accordance with the Preferred Reporting Items for Systematic Reviews and Meta-Analysis (PRISMA), (Page *et al*., 2021) and the Centre for Reviews and Dissemination guidance on the conduct and reporting of systematic reviews, (Akers *et al.*, 2009) and the protocol is registered at the International Prospective Register of Systematic Reviews (CRD42021229449) (Zavala *et al*., 2020).

### Search strategy

With input from an information specialist, we searched OVID MEDLINE, EMBASE, CINAHL and APA PsychoINFO databases from inception to 14 December 2020. Combining relevant keywords for (1) Population (SMI); (2) Type of study (Randomised control trials) and; (3) Outcomes (health risk behaviours and body weight). Further relevant studies were sought by citation searching (forwards and backwards) of the included studies and relevant systematic reviews. The results of the database and citation tracking reference searches were stored and de-duplicated in an EndNote library (Bramer *et al*., 2016).

### Selection criteria

We included randomised controlled trials assessing interventions for health risk behaviours in people with SMI in LMICs (using the World bank gross national income (GNI) classification) (Santos *et al*., 2007). Population: People aged ≥18 years with SMI, including severe depression with psychotic features, psychotic disorders and bipolar disorders where the diagnosis had been validated against diagnostic criteria, such as International Classification of Diseases (ICD) and Diagnostic and Statistical Manual of Mental Disorders (DSM) (Segal, 2010; World Health Organization, 2004). Intervention: Any intervention targeting weight reduction and health risk behaviour (i.e. tobacco use, poor sleep, and alcohol and illicit substance abuse). Comparator: No intervention, placebo, very brief intervention, usual care. We excluded non-randomised trials, and one arm interventions, since they are not recommended to measure efficacy and effectiveness of interventions (Evans, 2010).

### Outcomes

We included any outcome related to weight or health risk behaviour. For instance, *Weight gain:* Reduction in body weight or body mass index (BMI). *Tobacco use:* Self-reported abstinence with biochemical verification, including expired carbon monoxide (CO level of <10 ppm, salivary cotinine <15 ng/ml, urinary cotinine <50 ng/ml, or serum cotinine <15 ng/ml), and reduction in levels of expired carbon monoxide, cotinine and nicotine. *Alcohol abuse:* Alcohol abstinence, frequency of alcohol use, and quantity of alcohol use measured with any standardised and validated questionnaires. *Substance use (illicit drug and unprescribed medication):* Self-reported abstinence measured as any standardised and validated questionnaires. Biochemically-verified abstinence was recorded where available. *Sleep:* Sleep time, self-reported prevalence of bad sleep (less than 7 h or more than 9 h per day) and improvement in insomnia measured by any standardised questionnaire. *Quality of life*: measured by any validated scale and adverse events.

### Screening and study selection

The EndNote library was exported to Covidence (Melbourne, Australia) and de-duplicated again (Covidence, 2016). Titles and abstracts were screened for potential eligibility by two independent reviewers, discrepancies were resolved through consensus, and disagreements were resolved by a third independent reviewer (GZ). Full text of potentially eligible studies was retrieved and independently assessed for eligibility by two reviewers. Missing data to assess eligibility were sought by contacting the corresponding authors. The reason for exclusion was recorded. Discrepancies were resolved by consulting a third reviewer, who independently assessed the study (PM). For included studies, multiple reports from the same study were linked.

### Data extraction

Each record was extracted and reviewed by two of three independent authors using a pre-designed data extraction form. Discrepancies were resolved through consensus, and disagreements were resolved by a third independent reviewer (GZ). Missing data were requested from the study authors. The extracted information included: Study reference (authors name, year of publication), study population, country, setting (primary care, community, secondary care, mental health care), study design, intervention aim, number of intervention groups, description of the intervention, comparison intervention(s), duration of the intervention and outcome collection: short term (<6 months), medium term (6≤12 months), long term (12 months or longer); number of participants, participant demographics (age, gender, ethnicity, index of deprivation/social class where specified), participant diagnoses (including diagnostic criteria according to ICD and DSM) and baseline characteristics, primary outcome measure, secondary outcome measures, overall effect size/relative effect of intervention and funding source.

### Risk of bias

The methodological quality of the included studies was independently assessed by two reviewers (GZ, OT) with discrepancies resolved by a third reviewer (PM). We used the Cochrane Collaboration risk of bias tool 2.0, (Higgins *et al*., 2019*a*) which assesses the risk of bias in five domains: randomisation; deviations from intended intervention; missing data; outcome measurement; and selection of reported results. The overall risk of bias was defined as the worst risk of bias in any of the domains. However, if a study was judged to have ‘some concerns’ about the risk of bias for more than three domains it was judged as at high risk of bias overall (Sterne *et al*., 2019). To best capture the current state and quality of research in this field, studies were not excluded based on quality assessment, and thus all eligible articles were included.

### Data synthesis and analysis

We conducted a narrative synthesis of the findings from the included studies, structured around the type of intervention, target population characteristics and type of outcome (Pope *et al*., 2007). It was only possible to conduct a meta-analysis on the studies addressing cardiometabolic risk. We used a random-effects model and calculated the effect sizes using the pooled mean difference; where the study did not report the standard deviation for the mean difference we used imputation methods described in the Cochrane handbook (Enzmann, 2015; Higgins *et al*., 2019*b*). Although not originally planned in our protocol, we undertook a sensitivity analysis to pool the high risk of bias and low/middle risk of bias studies separately. This emerged as an important but unanticipated variable when we looked at study quality using the Risk of Bias instrument. The meta-analyses were conducted using the ‘metafor’ package in R (Vienna, Austria) (R Core Team, 2020).

### Comparison with high-income countries

To compare evidence generated in HICs compared to LMICs, we did a systematic search using the same key words excluding the LMICs search terms and including ‘review and meta analysis’. HICs were defined using the World Bank GNI classification (Santos *et al*., 2007). We extracted data from published systematic reviews looking at each specific risk behaviour (i.e. cardiometabolic risk, smoking, substance abuse and sleeping), and compared the number of available studies of LMICs with those of HICs (Fantom and Serajuddin, 2016). This search was not originally planned in the protocol and was performed to (1) provide a rough estimate of the interventions conducted in HICs, and (2) evaluate if the studies found in this review were also included in other reviews.

We also mapped the countries where trial evidence is available and the number of trials available per country. Maps were generated using the r worldmap package in R 4.1 (Vienna, Austria).

## Results

**Fig. 1.**
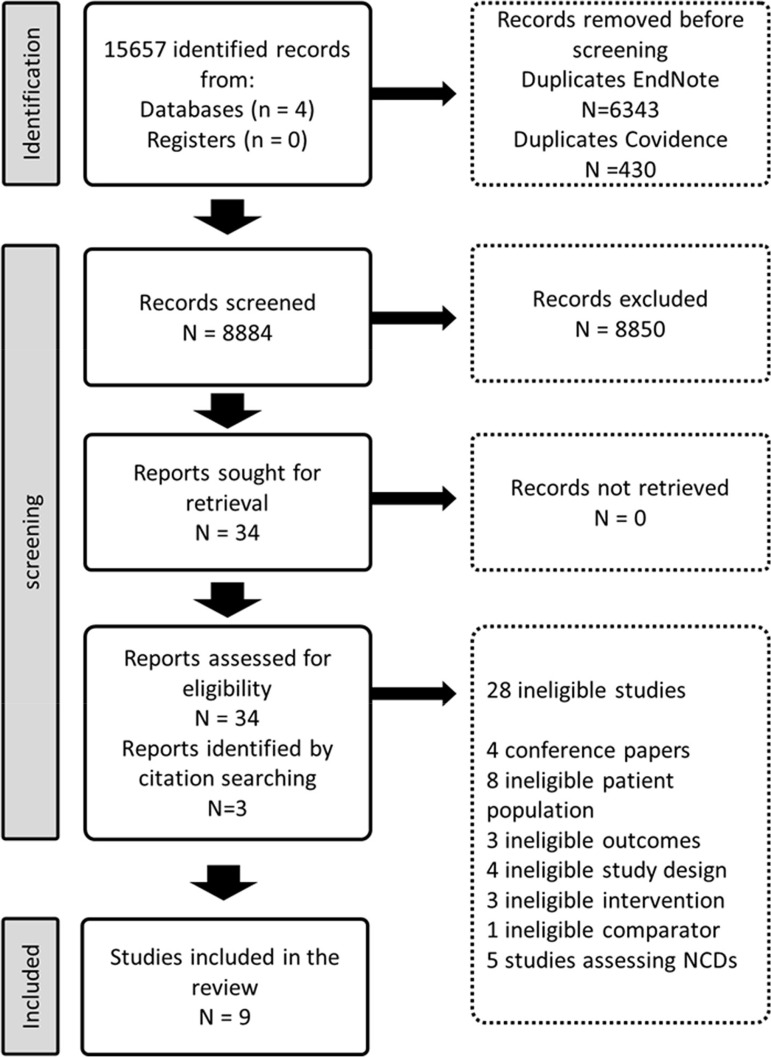
Identification of studies via databases, and references.

The search strategy identified 15 657 records. After removing 6773 duplicates, 8884 titles and abstracts were screened for eligibility (Fig. 1). We assessed the full texts of 34 eligible records from which 6 met the study inclusion criteria. We found three additional records from reference searching (Acil *et al*., 2008; Methapatara and Srisurapanont, 2011; Attux *et al*., 2013). Six trials focused on weight reduction, (Baptista *et al*., 2007; Wu *et al*., 2008; Methapatara and Srisurapanont, 2011; Attux *et al*., 2013; Romo-Nava *et al*., 2014; de Silva *et al*., 2015) (two lifestyle interventions, three pharmacological interventions and one was a combination of lifestyle and pharmacological interventions) (Wu *et al*., 2008; Methapatara and Srisurapanont, 2011; Attux *et al*., 2013), one on sleeping patterns (Kumar *et al*., 2007) and two on the effect of physical activity on quality of life (Acil *et al*., 2008; Loh *et al*., 2015) (Table 1).

**Table 1.** Main characteristics of the included studies

Author and country	Setting	Population (inclusion criteria)	Comparator	N	Type of intervention	Primary outcome(s)	Who delivered the intervention	Intervention format (individual/group/community)	Training and supervision (yes/no) By whom?	Fidelity measured (yes/no) and how?	Intervention duration	Follow up time	Overall ROB
Weight gain													
Attux *et al*. (2013) Brazil	Outpatients and inpatients	Schizophrenia DSM-IV 18–65 years Motivated or showing concerns about weight	Standard care	85	Lifestyle	Body weight BMI Waist circumference	Mental health professionals (nurses, occupational therapists, psychologists and dietitians)	Group (Patients and their relatives)	Yes, trained with a manual and a set of DVDs explaining the program Program coordinator supervises and follow up any absence in sessions	Not stated	12 weeks, 1 h sessions	12 weeks	Low
Baptista *et al*. (2007) Venezuela	Outpatients and inpatients	Schizophrenia (DSM-IV) <18 years Free of hormone replacement and any chronic diseases	Placebo	72	Pharmacological (Metformin 5–20 mg a day)	Body weight BMI Waist circumference	Research psychiatrists and social workers	Individual	Not stated	Not stated	12 weeks	12 weeks	Some concerns
de Silva *et al*. (2015) Sri Lanka	Outpatients	schizophrenia or schizoaffective disorder (ICD-10) <18 years treated with atypical antipsychotics, who had increased their pre-treatment body weight by more than 10%,	Placebo	66	Pharmacological (Metformin 500 mg a day)	Body weight BMI Waist circumference	Not stated	Individual	Yes, supervision – If there were problems with tolerability of 500 mg dosage, participants were treated with a lower dose of 250 mg, which was then titrated to the standard dose.	Not stated	24 weeks of treatment	24 weeks	Low
Methapatara and Srisurapanont (2011) Thailand	Outpatients	Schizophrenia (DSM-IV) 18–65 years BMI > 23 kg	Very brief intervention	64	Lifestyle	Body weight BMI Waist circumference	Therapist	Mixture of individual and group intervention	Yes, training on motivational interview	Not stated	6 sessions of 1 h each week	12 weeks	High
Romo-Nava *et al*. (2014) Mexico	Outpatients and inpatient	schizo-Phrenia or Bipolar disorder (DSM-IV) 18–45 years free of DSM-IV current substance abuse or a history of substance dependence in the last six months;	Placebo	44	Pharmacological (Melatonin 5 mg a day)	Body weight BMI Waist circumference	Psychiatrist	Individual	Yes, training of psychiatrists to deliver the intervention.	Not stated	Eight weeks of medication	8 weeks	Some concerns
Wu *et al*. (2008) China	Outpatients	18–45 years First psychotic episode of schizo-Phrenia (DSM-IV)	Placebo	128	Pharmacological and/or lifestyle intervention (Metformin 750 mg a day)	Body weight BMI Waist circumference	Physiologist	Individual for the pharmacological intervention, group for the lifestyle intervention	Not Stated	Not Stated	12 Weeks of medication or lifestyle interventions (psychoeducational, dietary, and exercise)	12 weeks	Low
Sleeping patterns													
Kumar *et al*. (2007) India	Outpatients	paranoid schizophre-nia.DSM-IV <18 years insomnia, defined as sleep-onset latency that was 30 min or greater, that had been present for at least the past 2 weeks	Placebo	40	Pharmacological (Melatonin 3–12 mg a day)	Time spent to fall asleep	Medical doctors	Individual	Not stated	Not stated	2 weeks	2 weeks	High
Physical activity													
Acil *et al*. (2008) Turkey	Outpatients	Schizophrenia DSM-IV	Usual care	30	Physical activity	World Health Organisation Quality of Life Scale-Turkish Version (WHOQOL-BREF-TR)	Psychiatrist	Group	Not stated	Not stated	10 week exercise program	10 weeks	High
Loh *et al*. (2015) Malaysia	Inpatients	schizophrenia between the age of 18 to 65 DSM-IV <18	Usual care	104	Physical activity (Structured walking)	Quality of life	Psychiatrist	Group	Yes, training was provided to therapists, nurses, psychiatrists, nutritionists, and pharmacists involved in the intervention.	Not stated	12 week exercise program	12 weeks	High

### Risk of bias

**Fig. 2.**
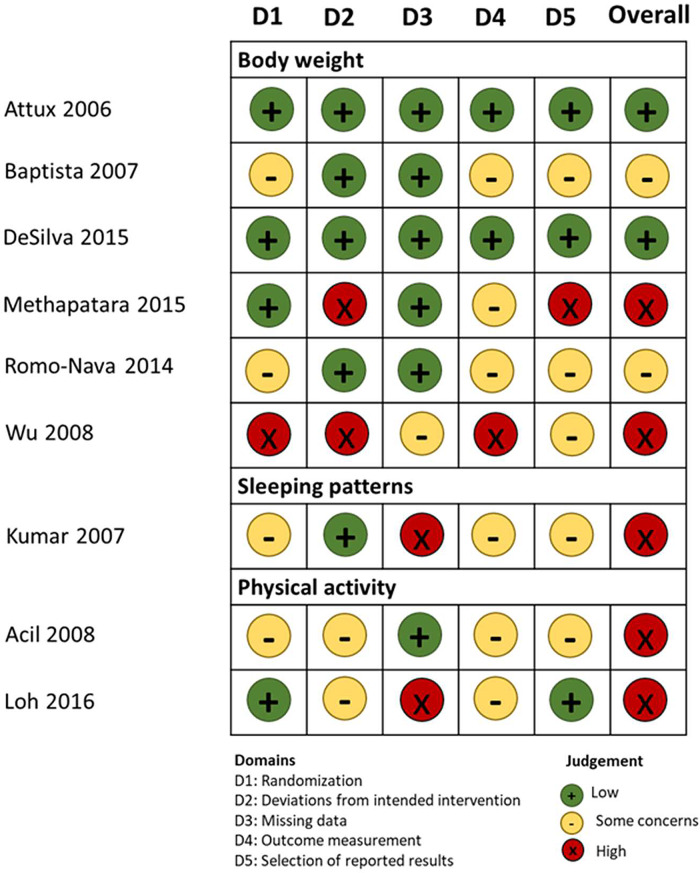
Risk of bias assessment of the eligible studies.

As seen in Fig. 2, two studies scored low in the overall risk of bias evaluation, (Attux *et al*., 2013; de Silva *et al*., 2015) two were evaluated as ‘with some concerns’ (Baptista *et al*., 2007; Romo-Nava *et al*., 2014) and five with high risk of bias (Kumar *et al*., 2007; Acil *et al*., 2008; Wu *et al*., 2008; Methapatara and Srisurapanont, 2011; Loh *et al*., 2015).

### Body weight

**Fig. 3.**
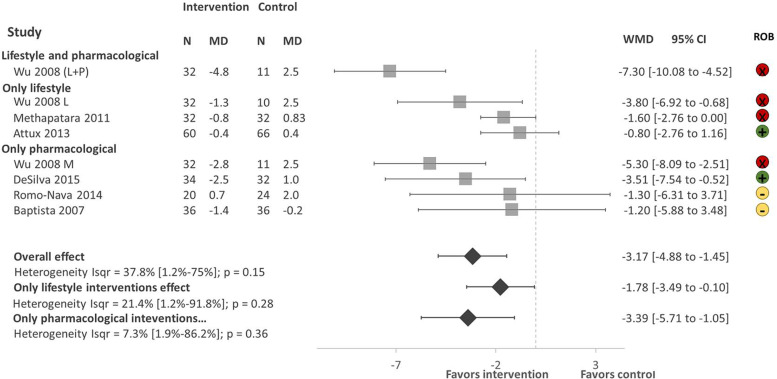
Forest plot of effect of lifestyle and pharmacological interventions on weight reduction in people with SMI. Effects on estimated weight reduction for each study depicted as solid squares; error bars indicate 95% CIs. The pooled estimates for overall effect, only lifestyle and only pharmacological interventions are shown as the diamonds. MD, mean difference between baseline and endpoint; WMD, weight mean difference between intervention and control; CI, confidence interval; ROB; Risk of bias; green, low, yellow, some concerns, red, high.

There were a total of 459 participants in the six studies, all focusing on weight reduction. All of the studies reported body weight, BMI and waist circumference as primary outcomes. As shown in Fig. 3. Lifestyle and pharmacological interventions alone and in combination are effective to reduce weight in people with SMI in LMICs with an overall pooled effect size of −3.17 Kg (95% CI −4.88 to −1.45, 6 studies, 500 subjects, *I*^2^ = 37.8%). The highest effect size was found on the combination of lifestyle and metformin (Wu *et al*., 2008) −7.30 Kg (95% CI −10.08 to −4.52, 1 study, 43 subjects), followed by pharmacological interventions with a pooled effect size of −3.39 Kg (95% CI −5.71 to −1.05, 4 studies, 225 subjects, *I*^2^ = 7.3%). We found low heterogeneity between the studies *I*^2^ = 37.8%.

### Sensitivity analysis

The sensitivity analysis excluding studies with a high risk of bias (Wu, 2008; Methapatara and Srisurapanont, 2011) included a total of 308 participants from four studies with a pool effect size of −2.21 (95% CI −4.89 to −0.47, *I*^2^ = 0.0%).

### Sleep

Only one study focused on sleeping patterns (Kumar *et al*., 2007). This study evaluated the use of melatonin to improve insomnia in people with schizophrenia. A total of 40 outpatients with DSM-IV paranoid schizophrenia were included in the study. The patients were not provided with behavioural counselling and prior psychotropic medications were continued and unchanged during the study in both groups. People in the intervention group had a significant reduction in the number of night time awakenings of 0.75 times (s.d. = 0.91) *v*. 1.70 times (s.d. = 0.57) in the control; increase in sleeping time, being 5.7 h (s.d. = 1.6) in the intervention group *v*. 5.4 h (s.d. = 0.9) in the control group. The use of melatonin was effective as a short-term hypnotic for patients with schizophrenia with insomnia, as participants who received the treatment experienced greater early morning freshness all through the study.

### Physical activity

Two studies focusing on the effect of physical activity on quality of life among chronic schizophrenia patients were found (Acil *et al*., 2008; Loh *et al*., 2015). The primary outcome measure was health-related quality of life measured with World Health Organization Quality of Life Scale-Turkish Version (WHOQOL-BREF-TR) in the study by Acil *et al*. (2008) and the SF-36 in the study by Loh *et al*. (2015) The study by Acil *et al*. (2008) showed that physical activity significantly improves all domains of quality of life (physical, mental, social, environmental and cultural), while the study by Loh *et al*. (2015) showed significant improvement on physical functioning, physical role and social functioning domains. It was not possible to conduct a meta analysis since the quality of life domains were different between the two studies.

### Cost-effectiveness

We could not find any evidence of cost or cost-effectiveness in any of the included trials

### Comparison with high-income countries

**Fig. 4.**
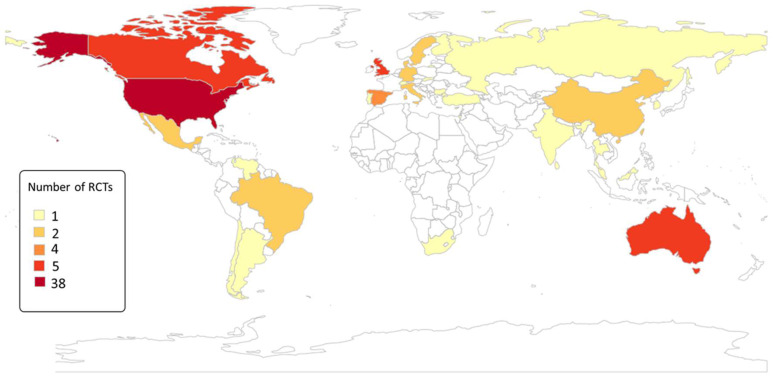
Number of RCT evaluating the effectiveness of interventions to address health risk behaviour in people with SMI.

As seen in Table 2, we found seven reviews assessing the effectiveness of interventions to reduce weight, perform physical activity and smoking in people with SMI. From the five systematic reviews, we identified 88 unique studies, of which only 6 were performed in LMICs (Fig. 4). In addition, five of the nine studies included in this review were not identified in the systematic reviews listed in Table 2.

**Table 2:** Systematic reviews assessing the effectiveness of interventions for body weight and health risk behaviour in people with SMI

Author	Title	Topic	Number of studies	Studies from LMICS
McGinty *et al*. (2016)^a^	Interventions to Address Medical Conditions and Health-Risk Behaviours Among Persons With Serious Mental Illness: A Comprehensive Review	Health-Risk Behaviours	43	2
Ashdown-Franks *et al*. (2018)	Is it possible for people with severe mental illness to sit less and move more? A systematic review of interventions to increase physical activity or reduce sedentary behaviour	Physical activity	16	1
Pearsall *et al*. (2014)	Exercise therapy in adults with serious mental illness: a systematic review and meta-analysis	Physical activity	8	2
Verhaeghe *et al*. (2011)^b^	Effectiveness and cost-effectiveness of lifestyle interventions on physical activity and eating habits in persons with severe mental disorders: a systematic review	Physical activity and diet	14	0
Naslund *et al*. (2017)	Lifestyle interventions for weight loss among overweight and obese adults with serious mental illness: A systematic review and meta-analysis	Body weight	17	1
Teasdale *et al*. (2017*a*, 2017*b*)	Solving a weighty problem: Systematic review and meta-analysis of nutrition interventions in severe mental illness	Body weight	20	1
Peckham *et al*. (2017)	Smoking cessation in severe mental ill health: what works? an updated systematic review and meta-analysis	Smoking	26	0

a Only the RCTs of interventions for weight management and smoking were included.

b Only RCTs were included.

## Discussion

### Main findings

We found only nine interventions targeting cardiometabolic and health risk behaviours in people with SMI from LMICs. We identified nine trials in nine LMICs and 88 trials across 20 HICs from other reviews. Overall we found that interventions to address cardiometabolic risk (focusing on weight reduction) and sleep were effective while there was a gap in trial evidence regarding smoking cessation and alcohol and illegal substance use.

### Interventions addressing cardiometabolic risk

All of the interventions focused on weight reduction and reported additional cardiometabolic risk factors as secondary outcomes. Similarly to our findings, evidence from HICs have shown that lifestyle and pharmacological interventions are effective to maintain and reduce body weight in people with SMI (Naslund *et al*., 2017; Teasdale *et al*., 2017*a*, 2017*b*). Despite the high prevalence of obesity in people with SMI, the known benefits of weight reduction, and the evidence of the effectiveness of lifestyle interventions, (Henry, 2011) people with SMI have consistently shown to have poor access to lifestyle interventions to reduce weight, specially in LMICs (Maj, 2009). In addition to the evidence of the effectiveness of pharmacological interventions to manage body weight, context-specific interventions evaluating the effectiveness of nutrition and physical activity interventions are needed in LMICs. People with SMI living in LMICs experience additional challenges such as less availability and affordability of healthy foods, walkability of the cities and safe spaces to perform physical activity (Kavle and Landry, 2018). Context-specific evidence could aid the development of programmes considering the challenges and barriers of people with SMI specific to their environment. (Teasdale *et al*., 2017*a*, 2017*b*)

### Interventions addressing health risk behaviours

Regardless of the high prevalence of health risk behaviours including physical inactivity, smoking, alcohol abuse, unhealthy diet, and poor sleep in people with SMI, (Prochaska *et al*., 2014; Vancampfort *et al*., 2017; Teasdale *et al*., 2017*a*, 2017*b*) trial evidence of the effectiveness of interventions to target these health risk behaviours in LMICs is limited or inexistent. This is further evidence of the health inequalities suffered by this population (Hallett and Rees, 2017). Adaptation of existing interventions might be a cost-effective approach to gather trial evidence in these settings, collect evidence on barriers and facilitators and provide information that may assist scaling up programmes promoting physical activity, smoking cessation, and improving the quality of sleep (Thornicroft *et al*., 2019).

### Information in HICs and LMICs

The disproportionate low number of trials in LMICs has been related to the low resources devoted to health care and insufficient funds to conduct research in these countries (Rathod *et al*., 2017). The majority of information comes from the USA, UK and Australia. While information in Africa, the Middle East and Latin America is nearly non-existent. Testing the feasibility and cost-effectiveness of context-appropriate interventions in these countries should be the first step to scale up larger programmes to address health risk behaviours to reduce inequalities, physical health conditions and the mortality gap in this population.

Most of the studies included in this review were missed by other recent reviews (published between 2011 and 2018). It is likely that we were able to identify them because of the specific search terms focusing on LMICs in our review and the additional databases we searched.

### Strengths and limitations

There are a few limitations of our study that require acknowledgement. (1) Most of the studies had small samples, and were categorised as having a high risk of bias. Furthermore, our sensitivity analyses show that these studies significantly bias the results of our meta-analyses. However, even after removing these studies with high risk of bias, the pooled effect on weight reduction remains statistically significant.; (2) There were minor deviations from the protocol. We intended to assess the effectiveness of interventions that focused on diet and physical activity for weight gain, however we also included pharmacological interventions with the same primary outcome (weight reduction). We did sub-group analysis according to the type of interventions, which allows for an assessment of non-pharmacological interventions independently. (3) We did not systematically search for the number of interventions to address weight and health risk behaviours in people with SMI in HICs, but systematically looked for available reviews that have looked in detail into these topics instead. There was an overlap in the RCTs included in the previously published reviews and our review, however, we excluded duplicated studies to avoid over-estimation of studies in any particular region. Despite these limitations, we provide for the first time a summary of the available interventions to address health risk behaviours and weight gain in people with SMI living in LMICs.

## Conclusion

Pharmacological and behavioural interventions were effective in reducing body weight in people with SMI. There was a limited number of interventions addressing sleep and physical activity and no interventions addressing smoking, alcohol or illicit drugs abuse. We found a disproportionate number of interventions performed in LMICs as compared to HICs. The absence of smoking cessation studies, and the gap between LMICs and HICs was the most surprising, since smoking makes the greatest contribution to poor health and health inequalities for people with SMIs. There is a need to test the feasibility and cost-effectiveness of context-appropriate interventions to address health risk behaviours and weight gain in people with SMI in LMIC, and to generate evidence that might aid in the development of policy and programmes to address these issues that might reduce the mortality gap in people with SMI.
